# Experimental study on failure characteristics and rheological properties of pillar-like rock samples with different shapes

**DOI:** 10.1038/s41598-023-46452-x

**Published:** 2023-12-16

**Authors:** Wen-bing Guo, Bi-bi Wang, Yi Tan, Gao-bo Zhao, Er-hu Bai, Ming-jie Guo, Peng Wen, Zhi-bao Ma, Wei-qiang Yang, Dong-tao Wu

**Affiliations:** 1 School of Energy Science and Engineering, Henan Polytechnic University, Jiaozuo, 454003 China; 2 Collaborative Innovative Center of Coal Safety Production in Henan Province, Jiaozuo, 454003 China; 3 West Virginia University College of Engineering and Mineral Resources, Morgantown, WV 26506 USA; 4 School of Environment and Resource, Southwest University of Science and Technology, Mianyang, 621010 China

**Keywords:** Energy science and technology, Engineering

## Abstract

The stability of coal pillar is extremely important to the control of rock strata movement and surface subsidence. It is of great significance for mining design to analyze the stability and failure characteristics of coal and rock pillars left after mining and to study the failure characteristics and rheological properties of coal and rock with different shapes. In this paper, based on uniaxial compression and rheological tests on rock samples, the rheological properties of rock samples with different shapes were discussed by using the nonlinear theoretical mechanics and damage theory, and the rheological mechanical characteristics of coarse yellow sandstone samples under the action of different free surface areas and the same loading contact area were investigated by means of experimental research, theoretical analysis and numerical simulation. The following conclusions were drawn: the failure characteristics and dynamic change process of rock samples with different shapes under the same loading contact area are obtained by uniaxial compression test and multi-stage rheological loading. The uniaxial compressive strengths of rock samples with the same loading contact surface area and different free surface areas are inversely proportional to their free surface areas. For the round sample, the stress level in the rheological test is obviously lower than the instantaneous peak uniaxial compression strength, while for the other samples, the stress level in the rheological test is close to the instantaneous peak uniaxial compression strength. For rock all these samples, both the ratio of steady-state rheological time to final failure time and the deformation degree decrease with the increase of free surface area.

## Introduction

As mining-induced environmental deterioration and geological disasters become increasingly severe, appropriate ways of reducing subsidence, controlling loss and realizing green mining have gained wide attention from researchers. The core of these problems is to control the movement of overburden left after, mining and reduce the damage to overburden and surface^1,2^. In order to prevent or reduce the related natural disasters caused by the rheological instability of coal and rock pillars of underground engineering such as surface subsidence, rock mass slide and ground instability, in-depth and long-lasting research has been conducted on the properties of rock mechanics and achieved many significant results in the aspects of strength, instability, deformation, damage, fracture and destruction.

Some researchers conducted laboratory tests to explore the mechanical characteristics of rock failure under true triaxial loading and unloading. For example, after performing true triaxial unloading tests, Li et al.^3^ concluded that the aspect ratio and intermediate principal stress of samples had an impact on the failure mode, peak strength and deformation degree of hard rock. Tomio Horibe^4^ concluded that under the condition of a certain loading contact area, the ultimate compressive strength of the material under different loads decreases with the increase of its perimeter (shape effect). Kong et al.^5^ carried out the true triaxial compression test on volcanic rocks and found that the brittle characteristics of rock failure are enhanced with the increase of intermediate principal stress. Moomivan, H. and Vutukuri, V.S. studied the effects of size and aspect ratio on the compressive strength of coal and rock samples^6^. Moomivan, H. studied the effect of size on the compressive strength of coal^7^. Moomivan, H. and Vutukuri, V.S. studied the effect of geometric shape on the compressive strength of columns^8^. These results have provided valuable insight into rock failure characteristics from the laboratory tests' perspectives.

In addition, the rheological properties of underground coal and rock pillars are extremely important for long-term stability and stratum control. Researchers all over the world have conducted extensive rheological test research on rock mechanical properties. For over a decade, Danesh, Fujii, Ladanyi, Ito et al.^9–12^ dedicated themselves to creep test research on the creep characteristics of coal and rock, as well as their influences on permeability and circumferential strain behavior of brittle rock. Chinese scholars Zhao and Yang et al.^13,14^ conducted creep tests and analysis on coal and rock under the influence of rock joints. Xia et al.^15^ developed and applied the full shear-seepage coupling of rock joints test system. Fu^16^ carried out experimental research on the size effect of uniaxial compressive strength of rocks with different height-diameter ratios. Xiao et al.^17^ carried out research on anisotropic creep characteristics of quartz-mica schist through triaxial compression creep tests. Meng et al.^18^ carried out experimental research on the influences of size effect and strain rate on rock mechanical properties. Under different circumstances, the stability of coal and rock will be affected by the surrounding geological conditions. Huang et al.^19^ researched on the creep damage mechanism of coal and rock and the stability of coal pillar on the gob side of the working face. However, the stability differences and failure characteristics of rock samples with different shapes and sizes are the problems that are rarely taken into consideration at present.

**Figure 1:**
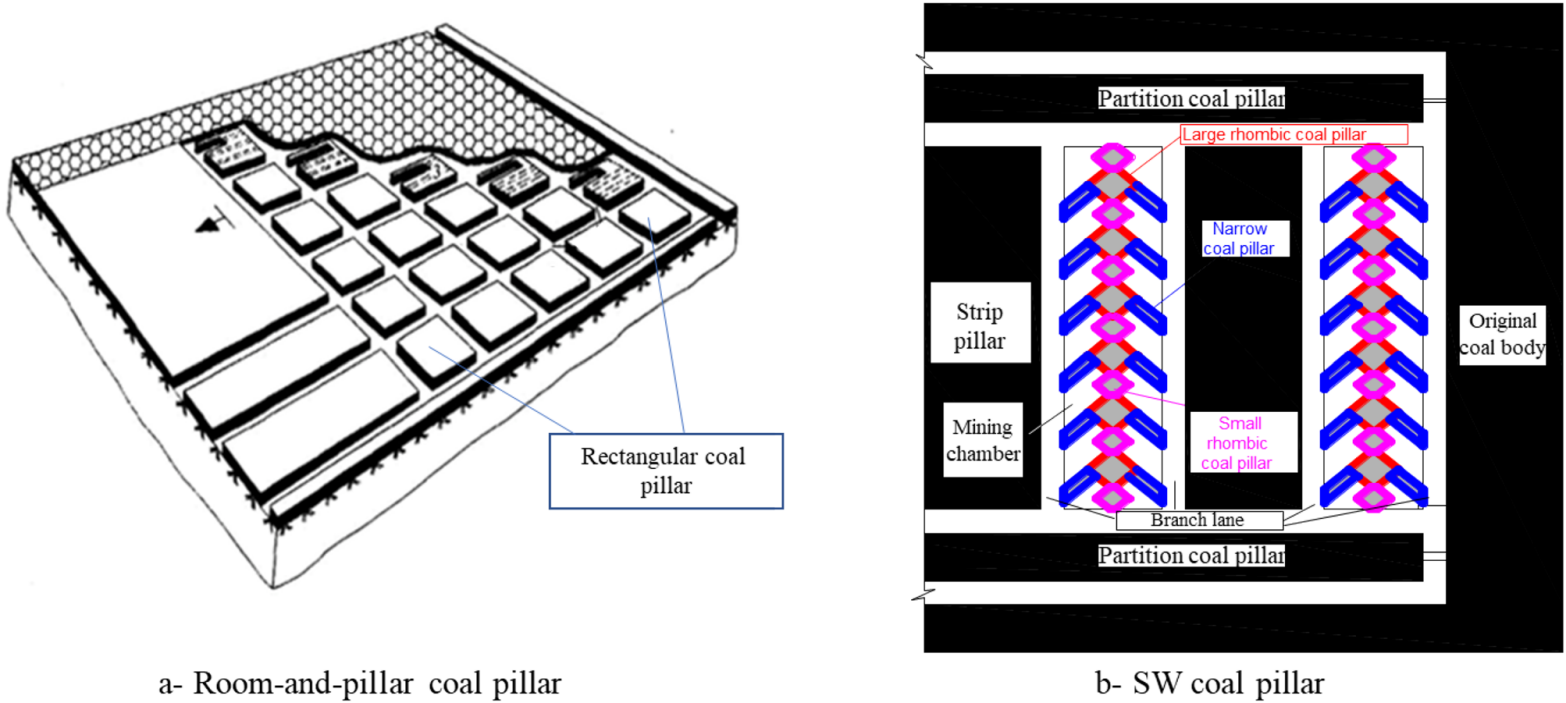
Shapes of coal pillars reserved under different mining methods.

Particularly, based on the concept of reducing subsidence, controlling loss, and realizing green mining, researchers have proposed the strip Wongawilli (SW) mining method^20^. This mining method leaves coal pillars with different shapes to control rock strata movement and surface subsidence. Guo et al.^20–22^ studied the design parameters of underground mining/excavation layout, working face layout, elastic–plastic zone of coal pillar, and stress state in the SW mining method through plentiful surveys and research, and drew useful conclusions. Particularly, the characteristics of reserved coal pillars under room-and-pillar mining are different from those under the SW mining method (Fig. 1). The patterns of coal pillars include rectangular coal pillars, strip coal pillars, rhombic coal pillars, narrow coal pillars, etc.

In this paper, the failure characteristics and rheological properties of samples with different shapes were investigated by means of experimental research, theoretical analysis and numerical simulation. Studying the failure forms, failure processes, and rheological characteristics of samples with different shapes can reveal the essence and laws of the failure of coal pillars with different shapes, providing scientific basis for the design and construction of mining engineering.

## Sample preparation and test system

### Sample preparation

According to the characteristics of reserved coal pillars under SW mining, the size of the single unit of the SW coal pillar, namely the irregular-shaped coal pillar, was scaled by an equal ratio of 1: 200. On this basis, the area of the loading surface of the rock sample was determined, and the sizes of the other five samples with different shapes were determined.

**Figure 2:**
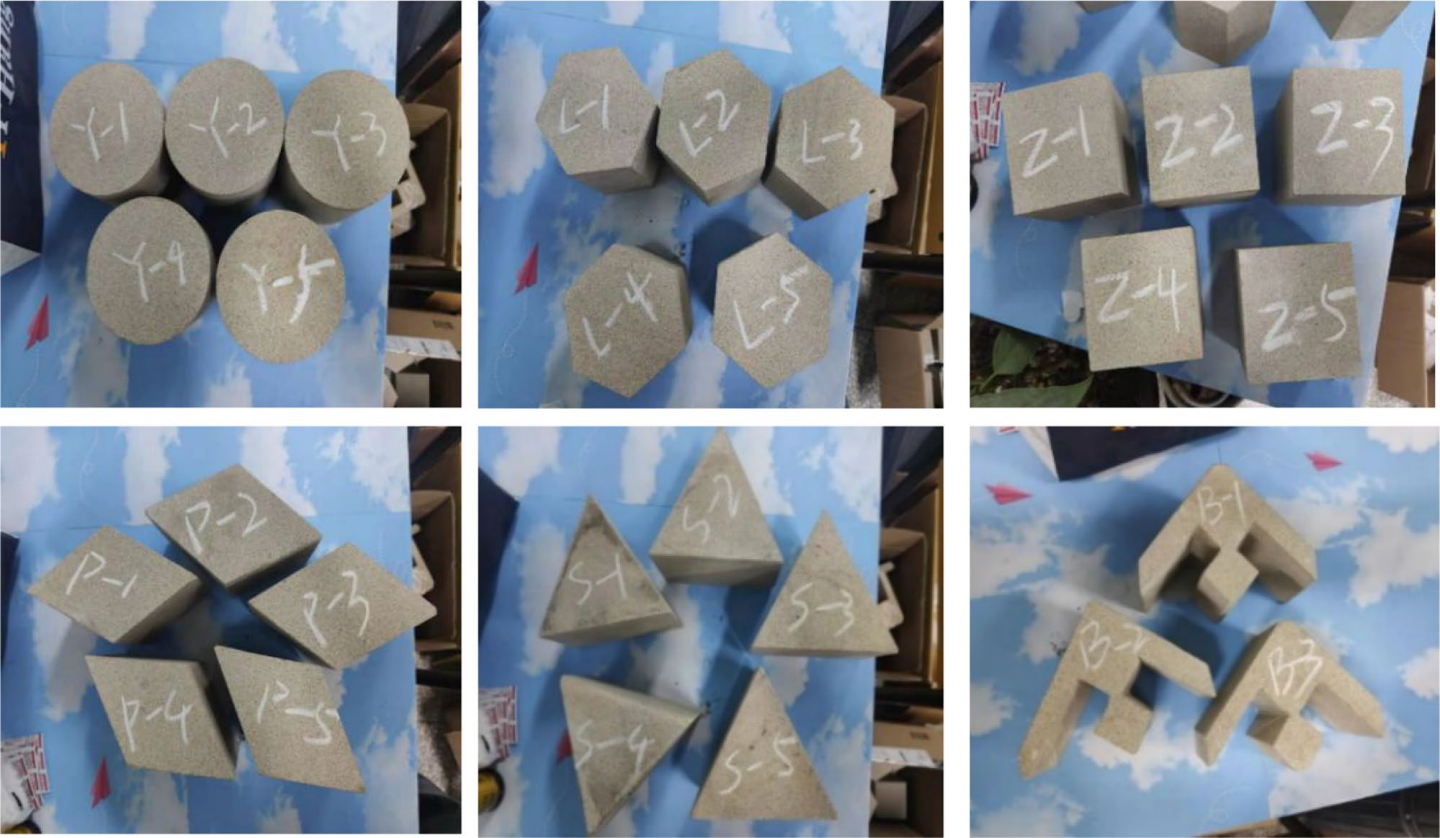
Finished products of columnar rock samples with different shapes.

Some collected coarse yellow sandstone blocks were processed by a RCD-250 drilling machine, a RLS-100 stone sawing machine, a RG-200 stone grinding machine and a line cutting machine in the laboratory into the following samples respectively: cylindrical samples with a diameter of 100 mm and a height of 100 mm, hexagonal prismatic samples with a side length of 55 mm and an angle of 120°, cubic samples with a side length of 89 mm and an angle of 90°, rhombic samples with a side length of 95.5 mm and an angle of 120°, triangular prismatic samples with a side length of 135 mm and an angle of 60°, and samples with the irregular shape (swallow shape). The loading surface areas of the samples with different shapes were all 0.0079 m^2^. The rock samples obtained by the above-mentioned machines are shown in Fig. 2.

The physical and mechanical parameters of rock samples obtained under standard sample size are listed in Table 1.

**Table 1:** Parameters related to cylindrical standard rock material (coarse yellow sand) (size: 50 × 100 mm).

Lithology	Water absorption rate/%	Uniaxial compressive strength/MPa	Elastic modulus/GPa	Deformation modulus/GPa	Poisson’s ratio
Coarse yellow sandstone	3.66	30.95	1.63	1.2	0.32

Conventional uniaxial compression and uniaxial rheological tests were conducted on coarse yellow sandstone samples with different shapes under the same loading surface area with the aid of an RLW-2000 rock triaxial rheometer and a DS2-16B acoustic emission tester.

### Test system

**Figure 3:**
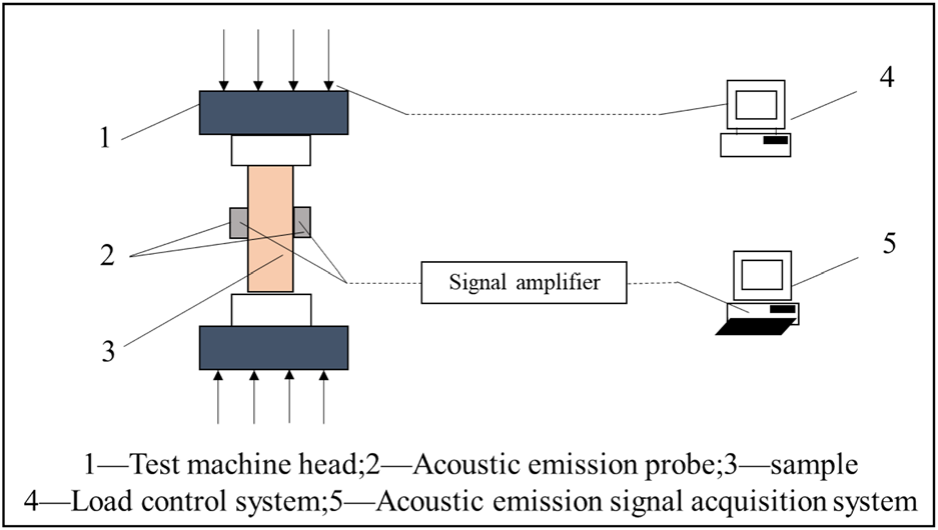
Rheological test system for acoustic emission and electromagnetic radiation signals from rock samples.

Uniaxial mechanical loading failure tests were carried out on samples with different shapes (cylinder, regular hexagon, square, rhombus, equilateral triangle and irregular Swallow shape) by the RLW-2000 rock triaxial rheometer (Fig. 3). The system, which is composed of a fully rigid mechanical loading system and a servo control and data acquisition system, can support a variety of stress path tests such as uniaxial tests, conventional triaxial tests, true triaxial tests, creep tests and cyclic loading and unloading tests.

### Acoustic emission testing system

**Figure 4:**
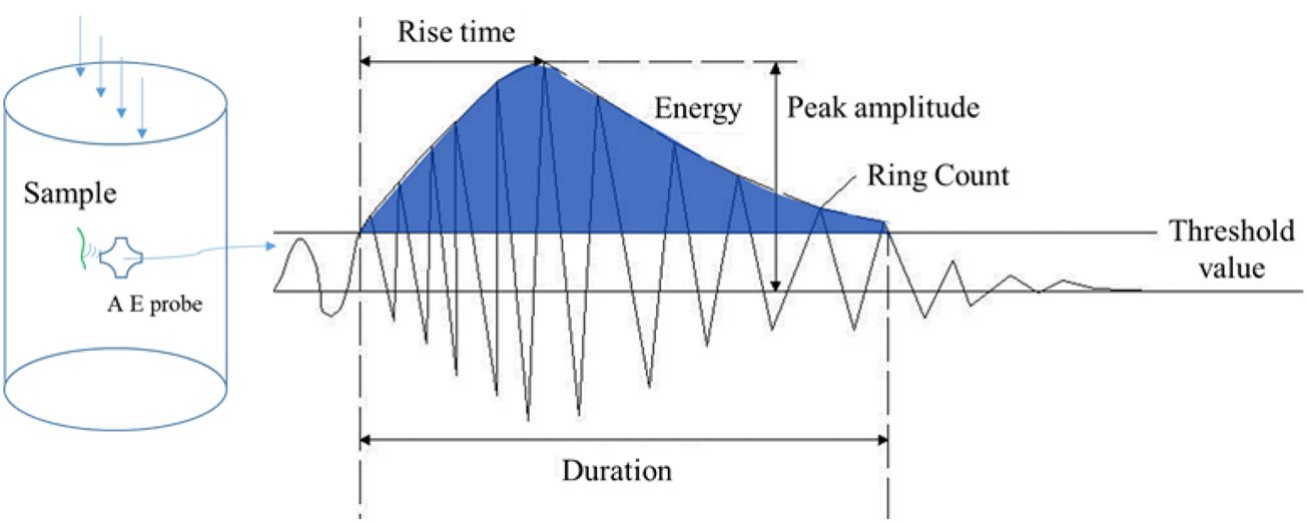
Characteristic parameter diagram of loading AE signal of coal pillar sample.

The acoustic emission monitoring system used during the experiment was from the DS2-16B eight channel acoustic emission signal processing equipment and eight AE Amplifier 40 dB acoustic emission sensors produced by Beijing Soft Island Times Company. (Acoustic emission frequency range: 50–400 kHz, center frequency: 150 kHz). The eight-channel acoustic emission signal interface of the DS2-16B acoustic emission detector system is placed on the different sides of the tested sample rock mass in the rock mechanics testing machine. The characteristic parameters of the acoustic emission signal loaded on the sample are shown in Fig. 4.

## Experimental study on failure of samples with different shapes under uniaxial instantaneous compression

### Uniaxial instantaneous compression failure experimental scheme

Each group of samples were loaded under an initial loading force of 30 KN by a loading rate of 158 N/s until they failed. The deformation degrees of the samples were collected by a digital dial gauge, and the bearing loads were directly collected and controlled by computer software.

### Failure characteristics of samples with different shapes under uniaxial instantaneous compression

**Figure 5:**
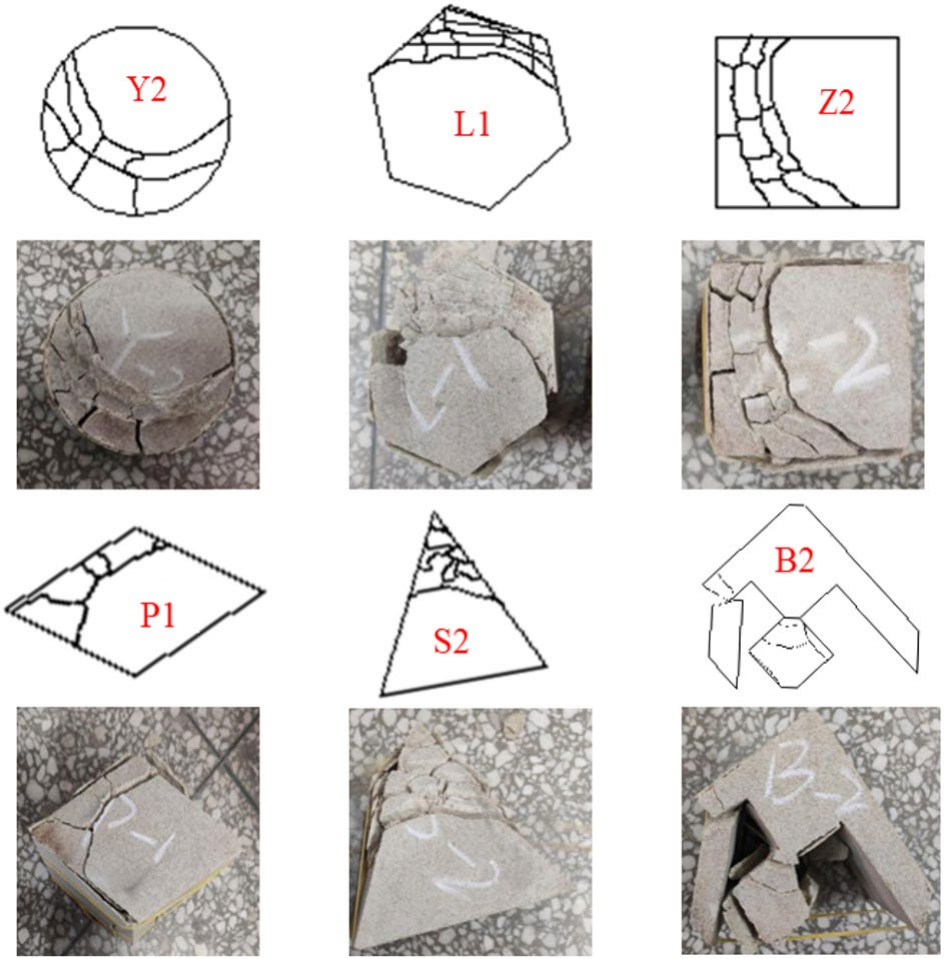
Failure characteristics of pillar-like rock samples with different shapes under uniaxial compression.

The morphologies and characteristics of samples with different shapes under the same loading contact area are shown in Fig. 5, from which the following points can be concluded.

1. When rock samples were loaded, the failure of the main shear surface mostly started from one end face and ended at the other end face, connecting both ends.
2. Some materials were crushed into powder at the stress concentration position during uniaxial compression instability failure by the testing machine.
3. The round sample also underwent tensile failure during loading failure, which was caused by cone shear failure at the end. A relatively complete cone could be taken out from the damaged sample.
4. The prismatic samples with sharp corners such as hexagonal, rhomboid and triangle were more likely to be destructed from the tip under the pressure of external load, and the smaller the angle, the greater the deformation degree.
5. Under the action of pressure, the sample with the SW special shape (swallow shape) often started to fail at the middle part of the swallow wing and the small diamond rather than at the sharp corner.

### Experimental analysis of samples with different shapes under uniaxial compression

According to uniaxial compression test results of samples with different shapes, their peak uniaxial compressive strength decreases with the increase of free surface (gob-side face). Meanwhile, for samples with different shapes, the uniaxial compressive strength ranges from 74.44 MPa under the free surface perimeter of 314 mm (round sample) to 52.49 MPa under 660.6 mm (swallow-shaped sample), as shown in Table 3.

**Figure 6:**
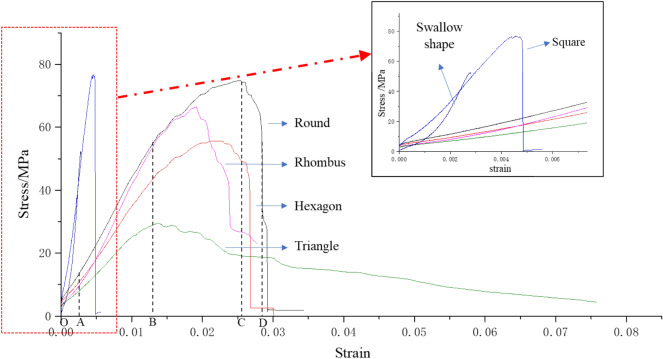
Stress–strain curves of pillar-like rock samples with different shapes (size: 100 * 100 mm).

As can be seen from the stress–strain curves in Fig. 6, the deformation of rock samples with different shapes can all be divided into four stages, i.e., the compaction stage, the elastic deformation stage, the nonlinear deformation stage and the strain softening stage. However, these stages present varying characteristics for different samples. Besides, the strain softening stage gradually lengthens with the increase of perimeter, that is, the larger the free surface, the larger the strain value.

The results suggest that the material is obviously brittle, and the stress drops rapidly after the load reaches the peak strength. The stress–strain curves of rock samples with different shapes correspond to different characteristics. Specifically, the stress–strain curve of the round sample approximates that of a conventional standard sample. Those of the square and swallow-shape samples are relatively incomplete, and these samples fail soon after the peak strength is reached, leaving a quite short strain softening stage. The rest three samples do not experience a sharp drop of stress when the peak strength is reached, but show plastic deformation. That is, the round, regular hexagonal, rhombic and triangular samples exhibit a certain brittle-ductility characteristics.

### Analysis of acoustic emission data from loading experiments on samples with different shapes

From the acoustic emission monitoring graph, it can be seen that during the loading process of the round specimen, the acoustic emission impact number shows a shape similar to the stress–strain curve, with several states, namely the stage of rapid increase in impact number, the stage of yield reduction, and the final stage of stable failure of the specimen. After being loaded for a certain period, the impact number of the rhombus specimen continues to increase, indicating that the specimen's accumulated energy and elastic energy are large. After a period of failure, the swallow-shaped specimen still has a certain load-bearing capacity in the middle, and then fails and becomes unstable again under the action of force. From the acoustic emission monitoring data of the triangular specimen loading, it was found that the failure of the specimen is a continuous process, from the initiation of the crack to the final instability. The failure of the specimen is a continuous steady-state process, indicating that the triangle specimen is the least capable of bearing capacity under force loading.

Besides, In the loading of regular rock samples such as round, hexagons, squares, and triangle, the monitoring activity patterns of each free surface acoustic emission channel are nearly similar. Based on the ringing counts and impact times of acoustic emission monitoring in each stage, different shapes can be loaded under the same load area. The data is different, but the difference is not significant, indicating that the shape effect (free surface effect) of regular rock samples is not significant; At the triangular loading acoustic emission monitoring station, it was found that the ringing count and number of impacts were relatively severe, indicating that the triangular sample is prone to damage; Especially in the acoustic emission monitoring of irregular (swallow shaped) specimen loading, it was found that the range values of each channel were very large. It is believed that the stress activity of each free surface of the swallow-shaped specimen under load is intense in the middle region of the swallow-shaped wing.

**Figure 7:**
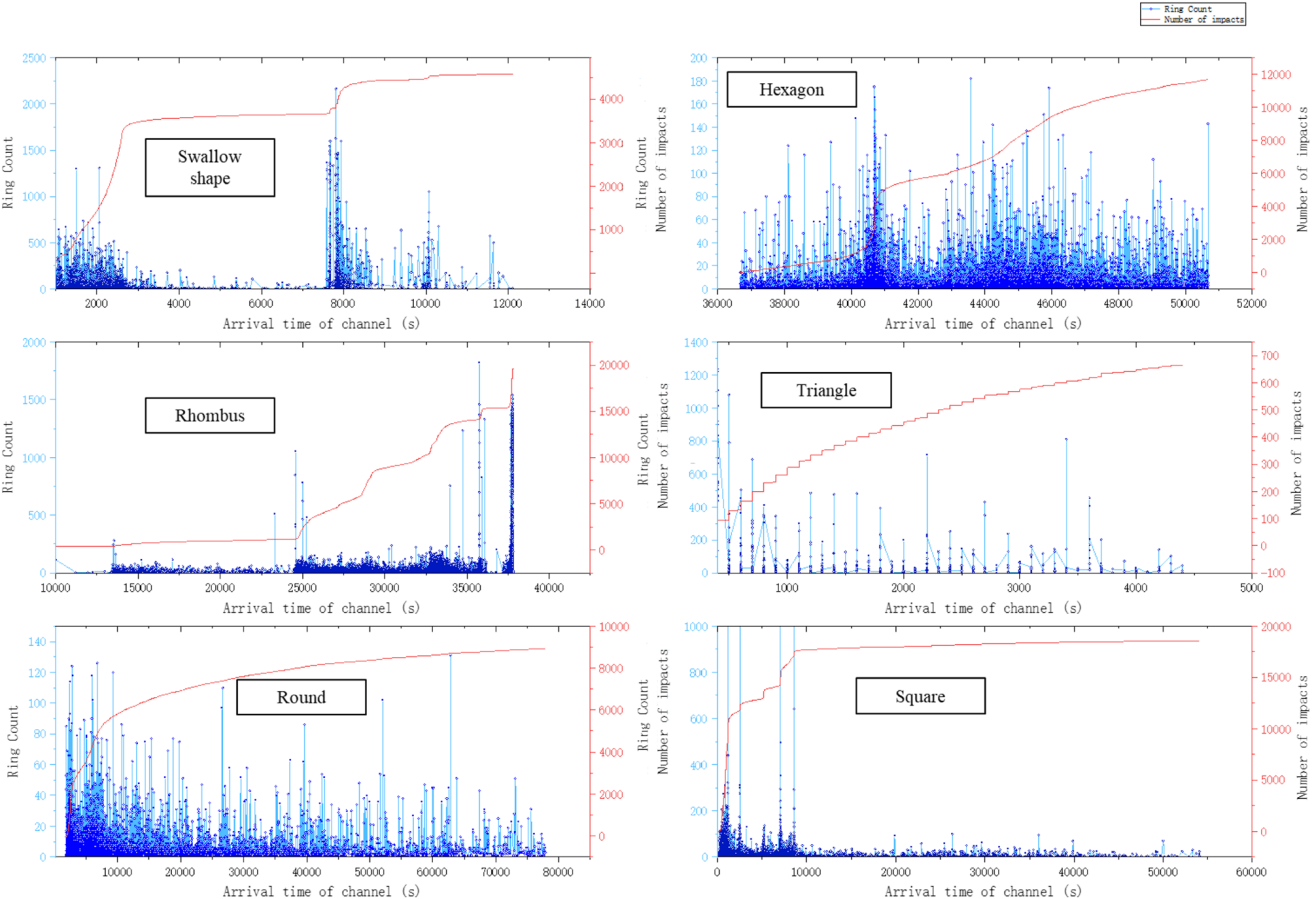
Acoustic emission monitoring data chart for loading different shaped specimens.

The acoustic emission test results are shown in Fig. 7.

## 3DEC numerical simulation on failure of samples with different shapes

### Selection of numerical simulation model and parameters

In the hope of better and more fully analyzing the shape (free surface) effect of the rock samples, Tyson polygons were constructed with the aid of third-party software Neper to simulate rock internal joints, and then the established rock models with different shapes were imported into 3DEC to simulate the instability failure properties of these samples.

3DEC is the abbreviation for 3 Dimension Distinct Element Code, which is the three-dimensional discrete element method program. As the name suggests, 3DEC is a computational analysis program based on the discrete element method as the basic theory to describe the mechanical behavior of discrete media. The Lagrangian solution mode determines that 3DEC has strong universality analysis capabilities in the field of continuous medium mechanics. At the same time, the core idea of the discrete element method endows 3DEC with essential advantages in dealing with non-continuous medium links, especially suitable for the analysis of static and dynamic response problems of discrete medium under loads (force load, fluid, temperature, etc.), such as the study of medium motion, large deformation, or failure behavior and failure process. 3DEC has a wide range of analysis modules in the field of geotechnical engineering, including dynamic analysis, rheological analysis, temperature analysis, joint network flow analysis, and other modules.

Tyson polygon is a subdivision of a spatial plane, characterized by the closest distance from any point within the polygon to its sample points (such as residential areas), the farthest distance from adjacent polygon samples, and each polygon containing only one sample point. Due to the bisection characteristics of Tyson polygons in space, they can be used to solve discretization problems. Neper is a software package for multi crystal generation and meshing. Polycrystals can be 2D or 3D. Neper is built around four modules: module T generates polycrystals as inlays; M module grid polycrystals are described as embedded files; Module S collaborates with FEPX to generate a simulation directory; Module V generates subdivision, mesh, and simulation results of PNG images or VTK files with publication quality (for interactive visualization).

**Figure 8:**
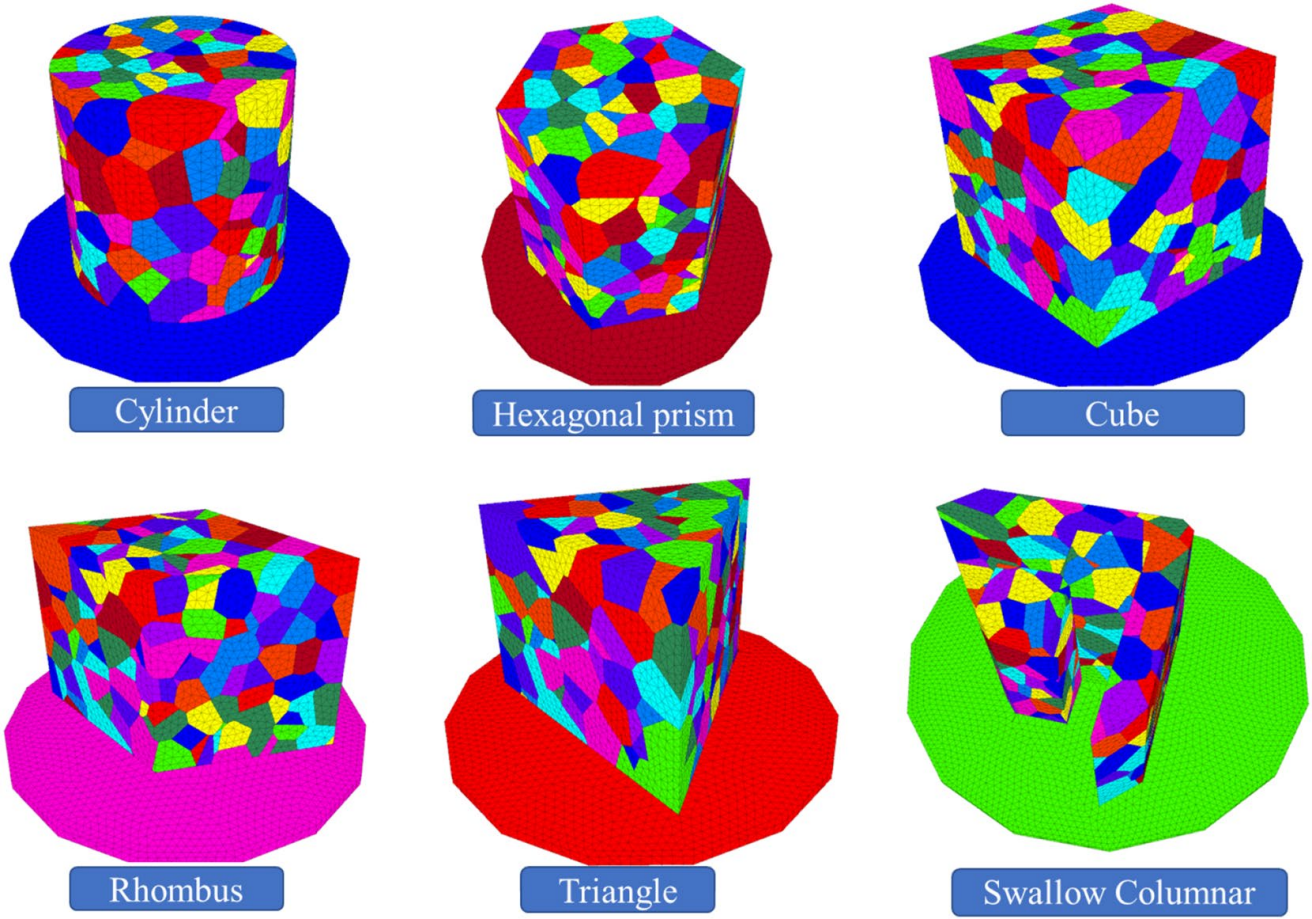
Construction of Tyson polygon models with different shapes.

The built model is shown in Fig. 8.

The selected values of numerical simulation parameters are given in Table 2.

**Table 2:** Parameters of numerical simulation^23^.

Contact	Elastic modulus/E(GPa)	Poisson’s ratio	Cohesive force/C(MPa)	Internal friction angle/Φ°	Normal stiffness (Kn) GPa/m	Shear stiffness (Ks) GPa/m
Joint of coarse yellow sandstone	19	0.20	19	38	4.2e4	4.2e3

The solving process of Normal stiffness (Kn) and Shear stiffness (Ks) is as follows :

1 $$G = \frac{E}{2(1 + v)}$$

2 $$K = \frac{E}{3(1 - 2v)}$$

3 $$Kn = factor \times \max \left[ {\frac{{\left( {K + \frac{4}{3}G} \right)}}{\Delta z\min }} \right]$$

where *E* is Young's modulus; *v* is Poisson's ratio; *K* and *G* are the bulk and shear moduli, respectively; *K*n and *K*s, are the normal stiffness and shear stiffness, respectively; factor is a multiplication factor(usually set to 10); △z_min_ is the smallest width of an adjoining zone in the normal direction,0.005 m.

### Numerical simulation results and analysis

**Figure 9:**
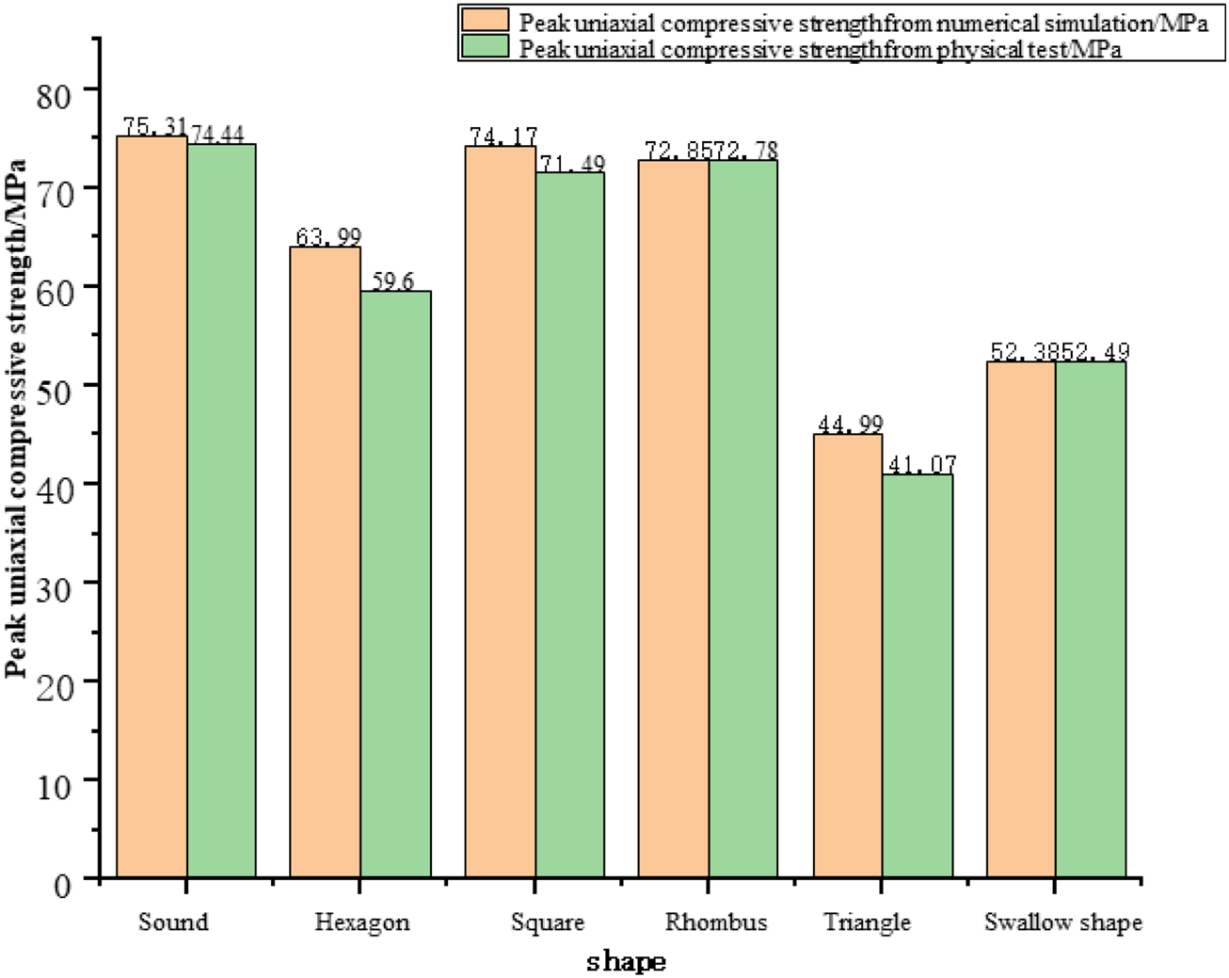
Comparison of peak strengths of pillar-like rock samples with different shapes obtained from 3DEC numerical simulation and physical test.

As given in Table 3, the uniaxial compressive strength of samples with different shapes obtained from the numerical simulation experiment ranges from 75.31 MPa of the round sample to 52.38 MPa of the swallow-shaped sample. The peak uniaxial compressive strengths of samples also show a rule of decreasing with the increase of free surface. The comparison of uniaxial compressive strength of different shapes obtained by different experimental methods is shown in Fig. 9.

**Table 3:** Uniaxial compressive strengths of samples with different shapes obtained from different experiments.

	Round	Hexagon	Square	Rhombus	Triangle	Swallow shape
Perimeter of free surface/mm	314.0	340.0	356.0	382.0	405.0	660.6
Uniaxial compressive strength from physical test/MPa	74.44	59.60	71.49	72.78	41.07	52.49
Uniaxial compressive strength from numerical simulation/MPa	75.31	63.99	74.17	72.85	44.99	52.38

The peak strengths of samples with different shapes were obtained by 3DEC numerical simulation. The peak strength shows a downward trend overall, but that of the sample with an irregular shape (swallow shape) increases instead. Moreover, under the same loading contact area, the bearing capacity of this sample is larger than that of the triangular one, which provides reliable data for the irregular-shaped coal pillars.

**Figure 10:**
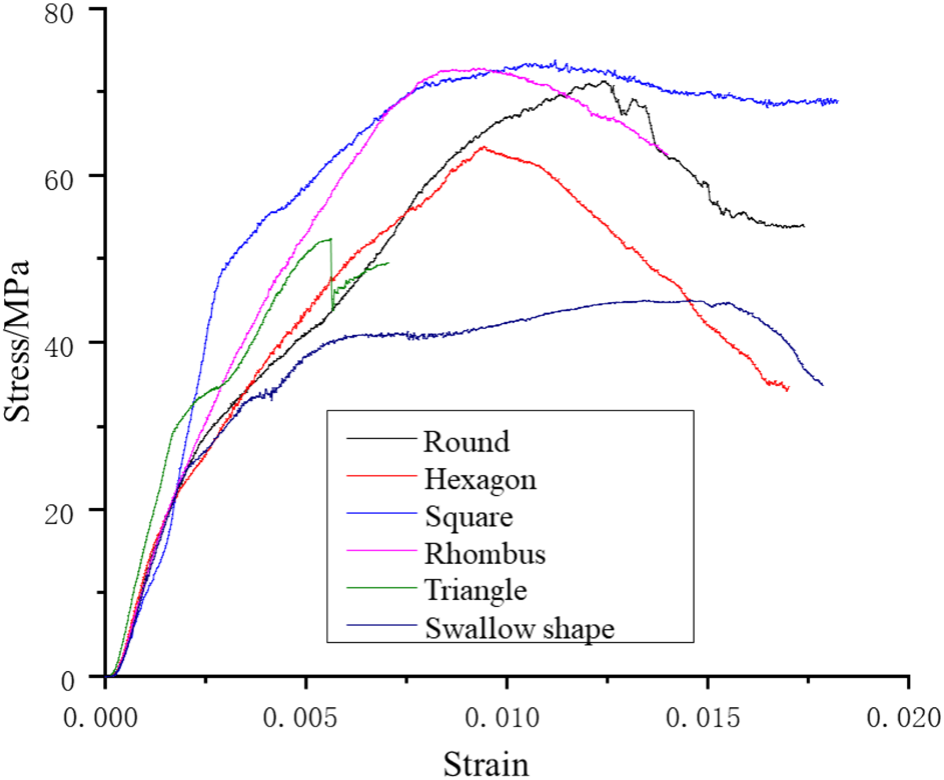
Stress strain curves of pillar-like rock samples with different shapes in 3DEC numerical simulation software.

The stress–strain curves of samples with different shapes obtained by 3DEC numerical simulation software are illustrated in Fig. 10.

**Figure 11:**
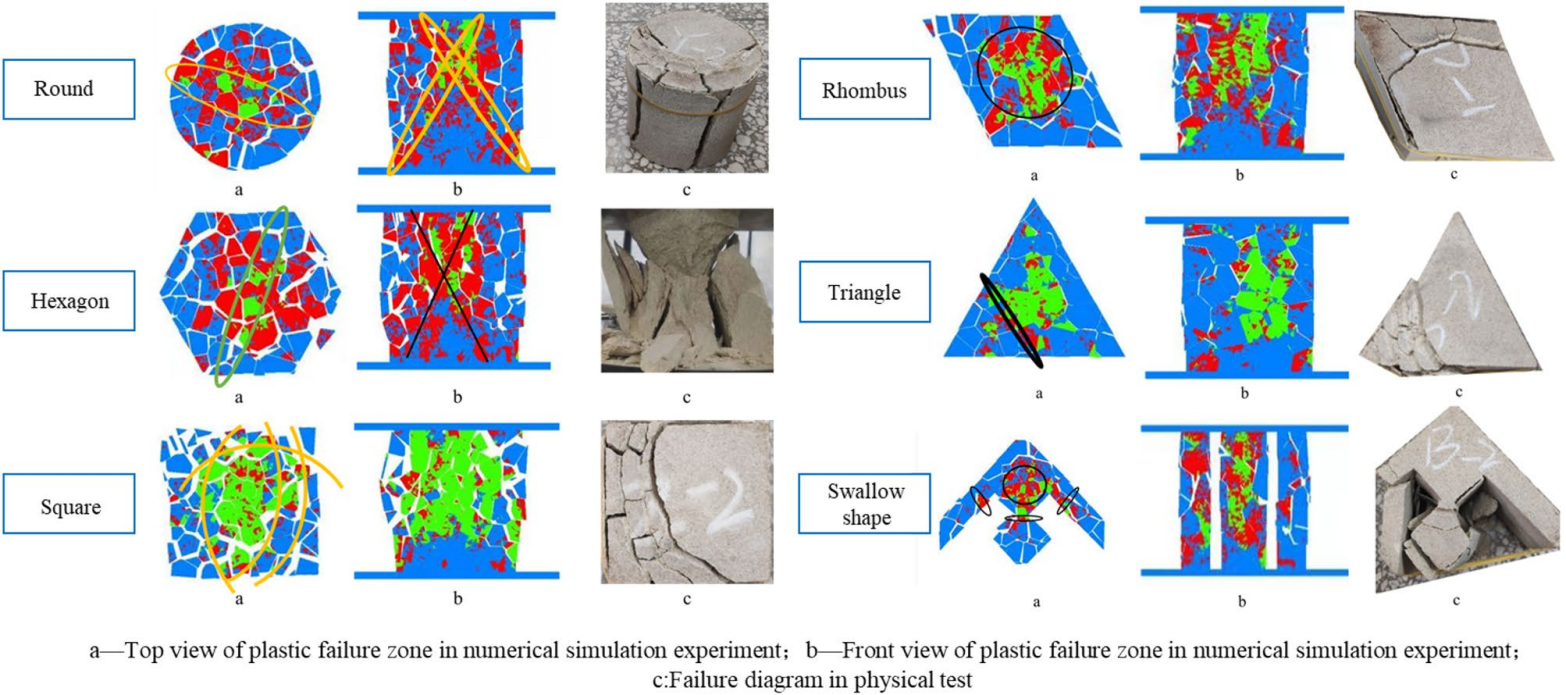
3DEC numerical simulation results and experimental results of plastic failure zones of pillar-like rock samples with different shapes.

The simulated uniaxial compression failure characteristics obtained based on the numerical simulation experiment of rock mechanics are presented in Fig. 11.

The following aspects can be seen in the simulated failure experiment on the samples with different shapes.

1. For the regular-shaped samples, the stress is usually concentrated at the center of the sample under the action of pressure, so that the failure develops from the middle.
2. The angular samples often start to fail from the edges of the prism, mainly due to the tip effect.
3. The round samples has the strongest bearing capacity, followed by the hexagonal, square and rhombic samples which have similar bearing capacities. It is noteworthy that the bearing capacities of the triangular and swallow-shaped samples are quite weak, of which that of the swallow-shaped sample is relatively strong.

## Experimental study on rheological instability properties of samples with different shapes

In order to obtain the rheological properties of rock samples with different shapes, rheological tests were performed on samples with six shapes mentioned above.

### Rheological test scheme

The test scheme is as follows.

1. Loading rate.
  After the pre-test and calculation, the rheological test was performed based on the force-loading control strategy, and the loading rate of the testing machine was set as 158 N/s.
2. Initial load.
  Considering the high strength of sandstone, the initial axial load was set as 30 kN.
3. Loading path.
  To study the rheological properties of samples in the high-stress area, loads that equaled 70%, 80%, 90% and 100% of their own ultimate strengths were applied in the first, second, third and fourth stages in turn until the samples failed finally.
4. Loading time.
  To study the steady-state behavior of specimens with different shapes, each stage of loading was maintained for 24 h.

### Rheological test results and analysis

**Figure 12:**
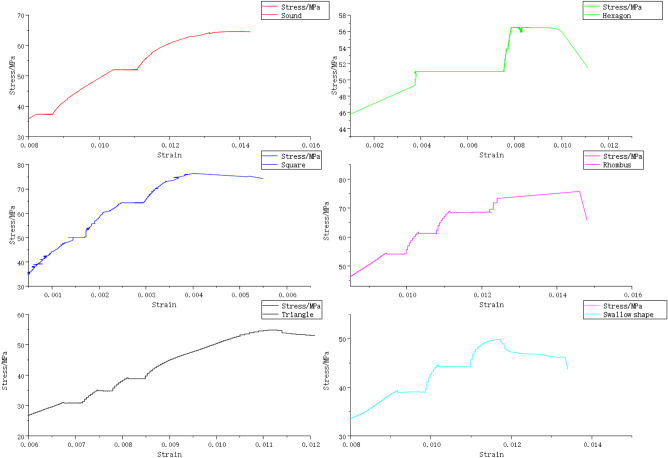
Stress–strain curves of pillar-like rock samples with different shapes subject to multi-stage loading by the RLW-2000 triaxial rheometer.

The following findings can be yielded through the stress–strain curves of rock samples with different shapes subject to multi-stage loading (Fig. 12).

1. Axial and tensile splitting failure of rock samples under uniaxial compression is not directly related to the reduction of their bearing capacities.
2. The rock samples with different shapes deform to varying degrees finally, and the deformation degree decreases with the increase of free surface area.

#### Experimental study on the rheological failure characteristics of samples with different shapes

**Figure 13:**
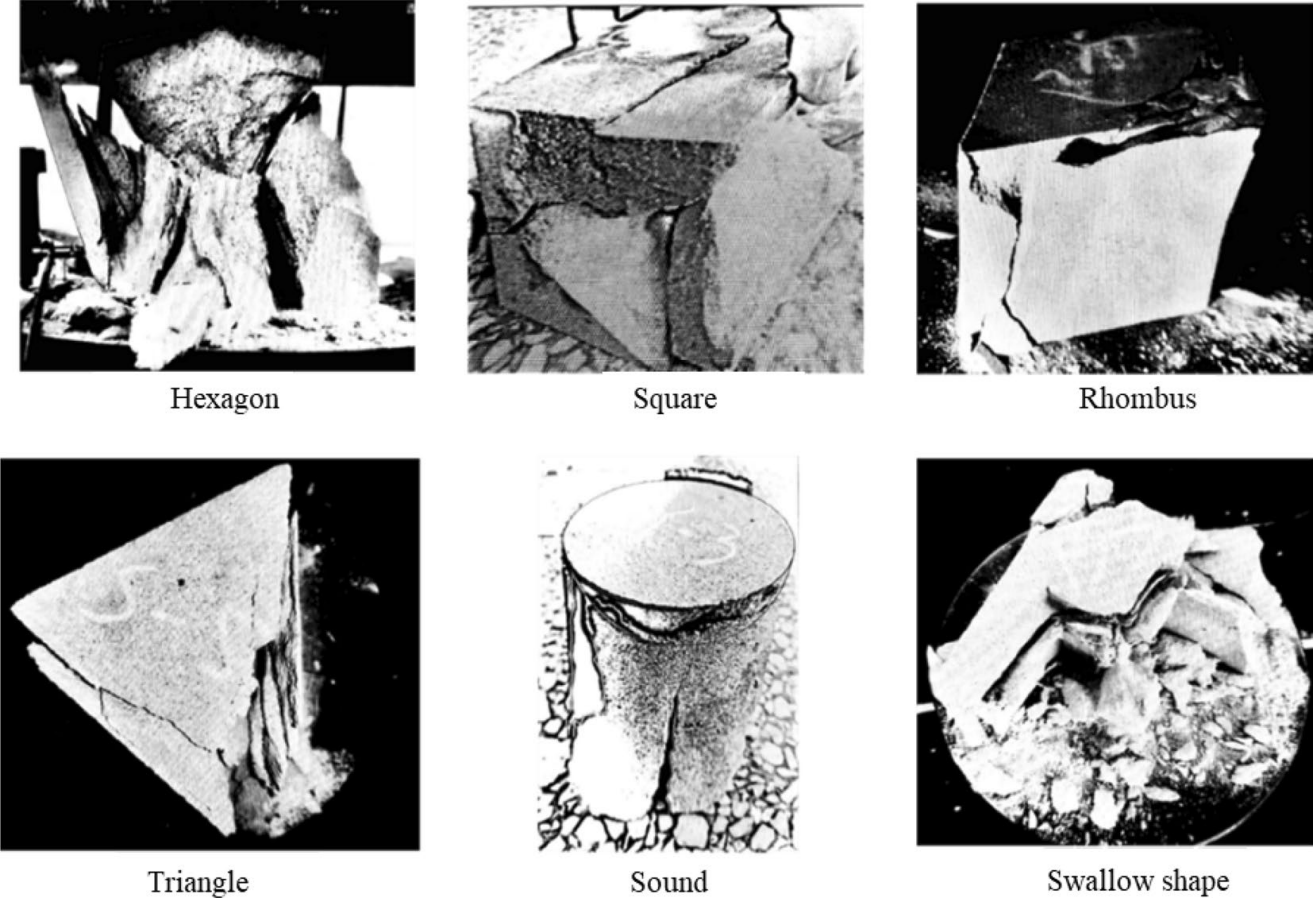
Rheological failure modes of pillar-like rock samples with different shapes.

Figure 13 exhibits the morphologies and characteristics of rheological failure of samples with different shapes under the same loading contact area. As can be observed from Fig. 13, when rock samples were loaded, the failure of the main shear surface mostly started from one end face and ended at the other end face, connecting both ends. Some materials were crushed into powder at the stress concentration position during uniaxial compression instability failure by the testing machine. The round sample also underwent tensile failure during loading failure, which was caused by the cone shear failure at the end. A relatively complete cone could be taken out from the damaged sample. The prismatic samples with sharp corners such as hexagonal, rhomboid and triangle were more likely to be destructed from the tip under the pressure of external load, and the smaller the angle, the greater the deformation degree. Under the action of pressure, the sample with the SW special shape (swallow shape) often started to fail at the middle part of the swallow wing and the small diamond rather than at the sharp corner. In short, with respect to failure mode, the coarse yellow sandstone samples with different shapes underwent brittle failure under the influence of pressure. In terms of failure mechanism, the round, hexagonal and square samples mainly showed compressive shear failure, while the rhombic, triangular and swallow-shaped samples presented tensile fracture or fracturing failure.

#### Analysis on rheological test results of samples with different shapes

For the purpose of describing the steady-state rheological stage in the process of shear rheological deformation, the steady-state rheological time and final failure time of samples with different shapes were counted (Table 4). It can be found from Table 4 that the stability of the rock samples weakens with the increase of free surface. Based on the mathematical theory, the bearing efficiency formula of free surface of coarse yellow sandstone samples with different shapes under the same loading surface is obtained:

4 $$Y = 0.58017 + \left( {\frac{0.10782}{{0.56823 \times \sqrt {\pi /2} }}} \right) \times e^{{[ - 2 \times ((x - 0.89648/0.56823)^{2} ]}}$$

where *x* is the ratio of loading surface perimeter; *y* is the ratio of steady-state rheological time to final failure time.

**Table 4:** Statistics of steady-state rheological time and failure time of samples with different shapes.

Shape	Steady-state rheological time	Final failure time	**M = t**_**1**_**/t**_**2**_** (%)**
	t_1_/h	T_2_/h	
Round	52	72	0.72
Hexagon	37	52	0.71
Square	48	70	0.68
Rhombus	52.8	80	0.66
Triangle	50	78	0.64
Swallow shape	31	53	0.58

t_1_ in the table represents the time when there was no failure during specimen loading; T_2_ represents the total loading time of the specimen in the case of final instability. The period between t_1_ and t_2_ is the time from the specimen's micro failure to the final instability.

**Figure 14:**
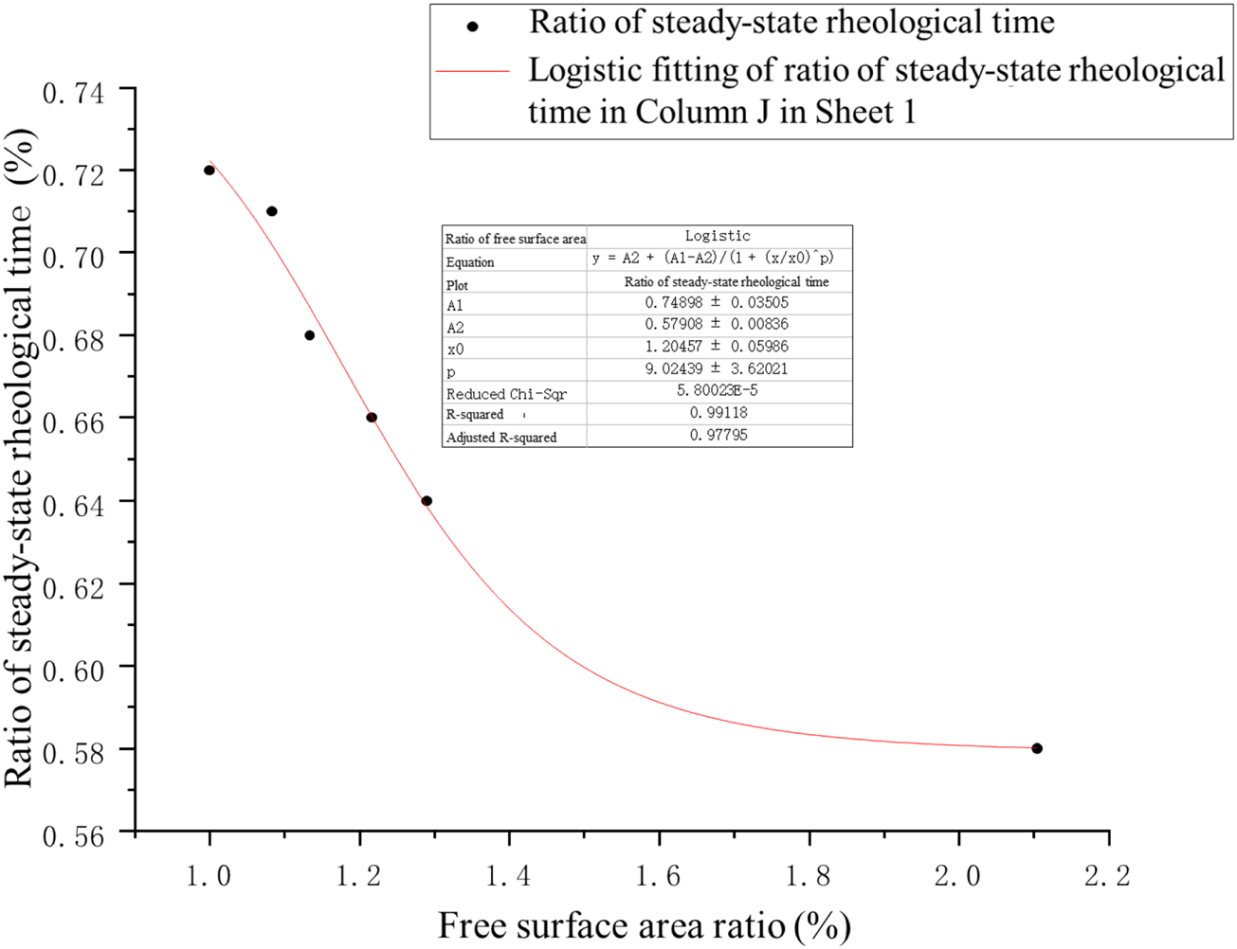
Relation curve between ratio of steady-state rheological time to final failure time and free surface area of pillar-like rock samples with different shapes.

As can be seen from the fitting curve in Fig. 14, when the free surface area (loading surface perimeter) accounts for over 20% of that of the round sample, the ratio of steady-state rheological time to final failure time no longer falls significantly. That is to say, the steady-state rheological time becomes similar regardless of the increase of free surface area after the ratio of free surface area exceeds 20%, which verifies the rationality of using swallow-shaped coal pillars in the SW mining method. This study guided the design of coal pillars in the continuous mining face of Wangtaipu Coal Mine and conducted a reliability analysis for the stability of the coal pillars left in the coal mine face.

**Figure 15:**
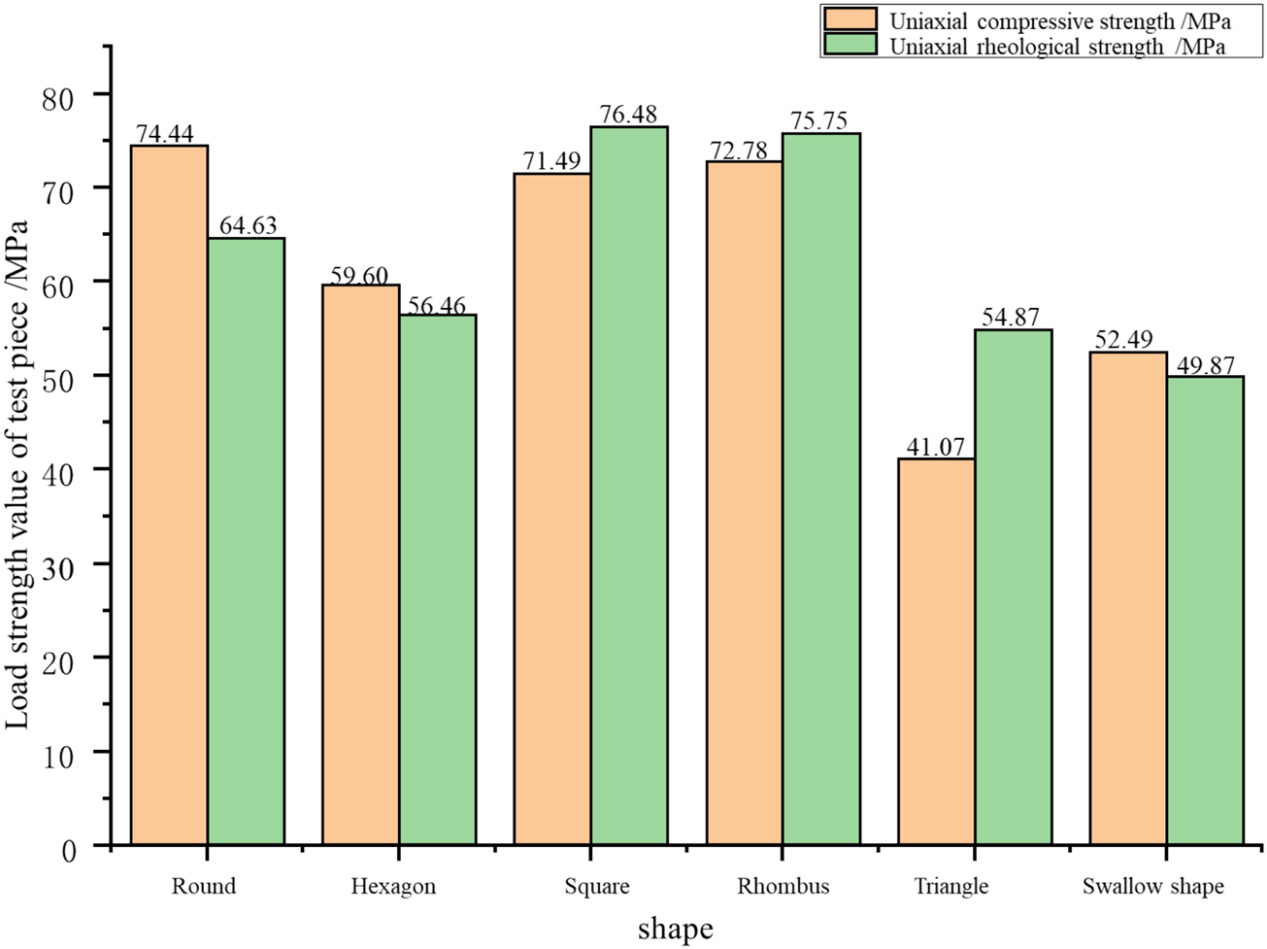
Comparison of peak strengths of pillar-like rock samples with different shapes under uniaxial compression and rheological conditions.

According to the uniaxial compression and uniaxial rheological compression test results of rocks with different shapes, the changes of peak uniaxial compressive strength under the two conditions are displayed in Fig. 15. Clearly, the peak uniaxial rheological strength of the round sample is far lower than its uniaxial compressive strength, while the peak uniaxial rheological strengths of the samples with the other shapes are close to their uniaxial compressive strengths.

## Discussion

In this paper, there are similarities and differences between the related research and the research of Tomio Horibe, Moomivand, H. The same point is that the compressive strength of rock samples with different shapes under the condition of regular shape decreases with the increase of free surface. The difference is that on the basis of the previous research, based on the special case of ' Tiaowang ' coal pillar, the swallow-shaped specimen test is added, and the best value of the free face space degree of freedom under the condition of the best stable proportion of coal pillar is obtained. This article analyzes the mechanical parameters of specimens of different shapes under the influence of different free surface areas under the same load surface area and a certain amount. The experiment considered the relevant effects under the same stress level. Next, we will study the failure characteristics and stress parameters of samples with different shapes under the same load stress conditions, as well as analyze the failure characteristics and physical and mechanical parameters of samples with different shapes under different moisture contents.

In the acoustic emission test results in Fig. 7a,c, there is a decrease in the number of impacts. This is the suspension of the acoustic emission probe during the loading process due to detachment from the sample. Reload and proceed. However, the overall trend of the ringing count does not affect the observation, that is, the damaged nodes during the experimental loading process can be accurately reflected.

## Conclusions

1. Rock samples with different shapes under the same loading surface area were prepared by the mode of SW coal pillars. Moreover, the ultimate peak strengths of samples with different shapes were acquired by uniaxial compressive strength loading tests. The results indicate that under the same loading surface area, the ultimate peak strength of regular-shaped rock decreases with the increase of free surface area.
2. The following findings were yielded through numerical simulation. For regular-shaped samples, the stress is generally concentrated at the middle of the sample, and the failure often start to occur there; for irregular-shaped samples, the angular samples often start to fail from the edges of the prism where the stress is concentrated; the swallow-shaped sample starts to fail from the two wings and the small diamond in turn before the failure develops to the whole sample. The effective bearing areas of samples with different shapes tend to decrease with the increase of free surface area. When the ratio of free surface area is more than 20%, the ratio of steady-state rheological time to final failure time of samples with different shapes no longer falls significantly.
3. According to the uniaxial rheological loading test results, the final deformation degrees of samples with different shapes differ, and the deformation degree decreases with the increase of free surface area.
4. In the analysis of the rheological test failure of rocks with different shapes, only the influence of particle flow was considered, and the effect of the similarity between coal (coal pillar) and rock (structural parameters such as water pressure and internal joints) on the failure of the specimen was not considered. In future research, it is necessary to study and analyze the single failure characteristics of the rock sample from the rich failure characteristics of coal pillars such as water pressure and weak joints.
5. This study on the rheological load of samples with different shapes only focuses on the percentage of ultimate load of each shape, without studying the rheological characteristics of samples with different shapes under the same load, which is the next step to be carried out.
6. In the numerical simulation analysis of the rock shape (free surface) effect, the influence of water pressure and confining pressure on the rock specimen was not considered, which is the focus of further research.

## Data Availability

The datasets used and analyzed during the current study are available from the corresponding author on reasonable request.
